# Selection and Optimization of the Parameters of the Robotized Packaging Process of One Type of Product

**DOI:** 10.3390/s20185378

**Published:** 2020-09-19

**Authors:** Szymon Borys, Wojciech Kaczmarek, Dariusz Laskowski

**Affiliations:** 1 Faculty of Mechatronics and Aerospace, Military University of Technology, Kaliskiego 2 Street, 00-908 Warsaw, Poland; wojciech.kaczmarek@wat.edu.pl; 2 Faculty of Electronics, Military University of Technology, Kaliskiego 2 Street, 00-908 Warsaw, Poland; dariusz.laskowski@wat.edu.pl

**Keywords:** industrial robot, packing, vision system, sorting, anomalous operation, system monitoring, sensors

## Abstract

The article presents the results of computer simulations related to the selection and optimization of the parameters of robotic packing process of one type of product. Taking the required performance of the robotic production line as a basis, we proposed its configuration using the RobotStudio environment for offline robot programming and virtual controller technology. Next, a methodology for the validation of the adopted assumptions was developed, based on a wide range of input data and a precise representation of the applicable conditions in the packaging process of one type of product. This methodology included test scenarios repeated an appropriate number of times in order to obtain the result data with the desired reliability and repeatability. The main element of the research was a computer simulation of the station based on the Picking PowerPac package. It was assumed that the products on the technological line are generated pseudo-randomly, thus reflecting the real working conditions. The result of the conducted works is the optimal operating speed of industrial robots and conveyors. The developed methodology allows for multifaceted analyses of the key parameters of the technological process (e.g., the number of active robots and their load, speed of conveyors, and station efficiency). We paid special attention to the occurrence of anomalies, i.e., emergency situations in the form of “halting” the operation of chosen robots and their impact on the obtained quality of the industrial process. As a result of the simulations, numerical values were obtained, maximum efficiency, with regard to maximum overflow of items of 5%, for LB algorithm was equal to 1188 completed containers per hour, with conveyors speeds of 270 mm/s and 165 mm/s. This efficiency was possible at robot speeds R1 = 6450 mm/s, R2 = 7500 mm/s, R3 = 6500 mm/s, R4 = 6375 mm/s, R5 = 5500 mm/s, R6 = 7200 mm/s. The ATC algorithm reached efficiency of 1332 containers per hour with less than 5% overflown items, with conveyor speeds of 310 mm/s and 185 mm/s. This efficiency was possible at robot speeds R1 = 7500 mm/s, R2 = 7500 mm/s, R3 = 7200 mm/s, R4 = 7000 mm/s, R5 = 6450 mm/s, R6 = 6300 mm/s. Tests carried out for emergency situations showed that the LB algorithm does not allow for automatic continuation of the process, while the ATC algorithm assured production efficiency of 94% to 98% of the maximum station efficiency.

## 1. Introduction

The robotics market in recent years has been developing at the level of about 15% annually [1]. Among the fastest growing branches of industry is the food industry, which is particularly demanding in the process of robotization due to the often irregular shapes of products and the need for frequent modifications of the production line [1,2]. Due to the high competitiveness of the market and the need to meet rigorous product quality standards, the strategies of the factories must be constantly changing; therefore, it is necessary to conduct analyses concerning current production [3]. On the other hand, due to a large number of technological and equipment parameters, achieving high productivity while ensuring quality is a big challenge [4,5]. Taking into account the high costs of robotic production lines, it is necessary to develop flexible production systems that will allow for quick adaptation to customers’ needs. This solution is possible thanks to the use of techniques and tools for the analysis of the current technological process with the possibility of predicting future functions of the system [6,7,8,9,10,11,12]. It is a good habit, when estimating the requirements of the process and creating behavioral models of both the process and individual machines, to consider possible problems and include them in this model. Given the high costs of conducting such experiments on the production floor, it makes sense to conduct computer simulations as early as at the production design—this allows for eliminating a number of problems. It is particularly important to carry out analyses that will allow the effectiveness of the robotic production process to be assessed in a numerical manner or will allow for assessing the scope of changes introduced [13]. Full analyses should consider the maintenance, planning, and sequencing of tasks, taking into account at the same time the stochasticity of processes and uncertainty related to the demand, supply, and performance of machines [14]. A different approach is a process analysis focusing on the time needed to complete each task and, in relation to it, the time needed to complete the entire order [15]. Despite the possibility to perform computer simulations at the production start-up or during production, it remains a difficult task to prepare a model based on the actual process [16].

**Figure 1:**
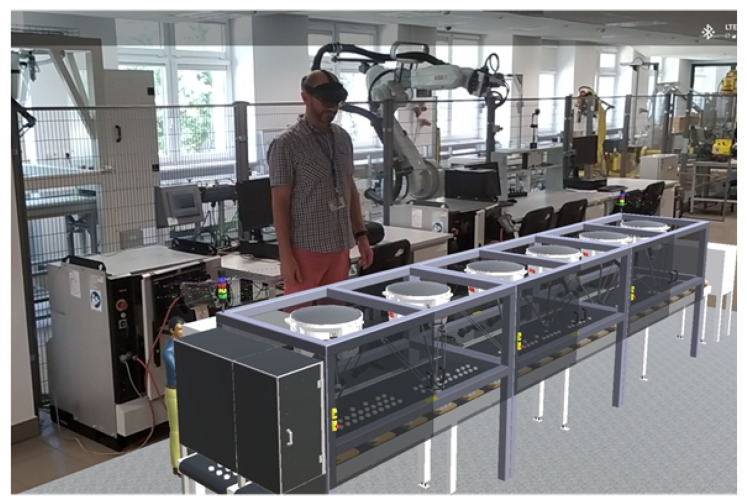
General view of prepared robotic cell using AR.

New challenges posed by Industry 4.0 have resulted in a dynamic development of simulation environments (e.g., RobotStudio, Robo Guide, KUKA SimPro, Delmia, Simulate Process) allowing for an accurate simulation of conditions on real production lines thanks to virtual controllers [17,18,19]. Creating digital twin manufacturing cells (DTMC) is possible using offline robot programming techniques that are being currently developed [20,21,22,23,24,25]. This makes it possible to carry out analyses of the functioning of production stations before they are put into operation, but what is also important is that it allows (quickly and without stopping the real machines) for modifications to be made during production. This is particularly important in the current trend of production personalization [26]. Digital twins of production cells can be used to investigate the strategy of collaboration between industrial robots and humans [27]. This approach increases operator safety related to the hazards of high-speed movements and massive forces generated by robots, and the need for humans and industrial robots to share a common workplace. Digital twins have one more important feature—they allow for creating advanced control applications with the use of the most modern tools, i.e., artificial intelligence (AI), virtual reality (VR), and augmented reality (AR) [20,28]. These technologies are also used in Robotics Laboratory at Military University of Technology in Warsaw. An example of AR, connecting HoloLens 2 with RobotStudio software, is shown in Figure 1.

In order to be able to accurately replicate reality, it is required that models of machines (including robots) are complete. It means that, for industrial robots, it is necessary to develop accurate kinematic models and control systems [29].

Due to the dynamic development of robotization of the electronics and food industries, pick and place applications are being developed to help solving the problems encountered in this type of robotization (complexity of the process, cooperation of people and the machines, irregular shapes of products) [1,30]. One of the important aspects in pick and place applications is planning the trajectory of manipulator movement, especially when using dual-arm robots [31,32,33,34]. The distribution of tasks among many cooperating robots is also a challenge, especially if they work on the same line [35]. It is necessary to coordinate machines properly and to queue and divide products between cooperating robots. Nowadays, vision systems are an integral part of pick and place applications. They provide high flexibility of the process and quick response of robots to production changes on the line. One of the most important problems concerning robots’ cooperation with vision systems is the accuracy of transmitted coordinates and orientation of detected objects [36,37,38]. In pick and place applications, where robots are expected to perform short work cycles, it is important to set appropriate time and gripping method [39]. Most of these grippers are suction type, which are required to have a gripping time of less than 1 s. An equally important parameter is the grasp reliability, which is crucial for fast and dynamic manipulator movements [40,41].

The effectiveness of a robotized workplace affects not only economic aspects but is also an important qualitative indicator of the company’s profitability. The highest possible number of manufactured products, in the shortest possible time with the highest possible quality are the main goals set by manufacturing companies. This process is measured by work cycles performed by specific robots in each period. Based on these cycles, it is also possible to find out whether the machines are working properly with the required repeatability. In case of packaging applications, the possibility to continue production in case of emergency situations should also be considered [42]. It may happen that, due to the failure of one machine (e.g., industrial robot), it is necessary to stop the whole production line. Such a situation generates huge losses, so we are looking for solutions that will ensure continuity of production even if it is not at 100% efficiency.

On robotic production lines, it is often necessary to balance and sequence tasks performed by machines (e.g., industrial robots). The division of tasks between machines is related to technological operations that are necessary to realize a given production process. Due to the complexity of the assembly processes, it is particularly noticeable on assembly lines (e.g., in the automotive). However, it is easy to notice that the robotization of other production processes (e.g., packaging, sorting, arc welding, spot welding, cutting) is guided by the same principles. This is evident if there are many robots working in a cell or production line, often working together to achieve the goal. Therefore, designing technological lines is not an easy task for manufacturers. Designers must take into account many factors (e.g., factory layout, cost, and reliability of the system, complexity of the tasks, equipment selection, line operating criteria, constraints) that affect the end result. A wide range of solution procedures can be taken into consideration. Research papers specify:

- heuristic methods that include constructive procedure, genetic algorithms, tabu search, simulated annealing, and ant colony optimization [43,44,45,46],
- optimum seeking models (branch and bound search, dynamic programming, linear, and integral programming) [47,48],
- other approaches (theory of constrains, knowledge-based approach, expert systems) [49,50,51],
- simulation methods [52,53].

The designer’s aim is to develop a line that considers high productivity, optimized processing time, cost effectiveness, less balance delays, flexible production, and just-in-time production. It is crucial to propose lines by using the best design methods. In our considerations, we used simulation methods, which guarantees obtaining reliable test results. Advanced environments for programming robots and simulating the work of robotic cells (e.g., RobotStudio) available on the market allow for accurately reflecting real conditions (according to the producers, such technology offers 99% replication). This is possible thanks to the use of the virtual controllers created on the basis of a real robot systems. This technology allows the development of digital twins that are equivalent to real machines.

**Figure 2:**
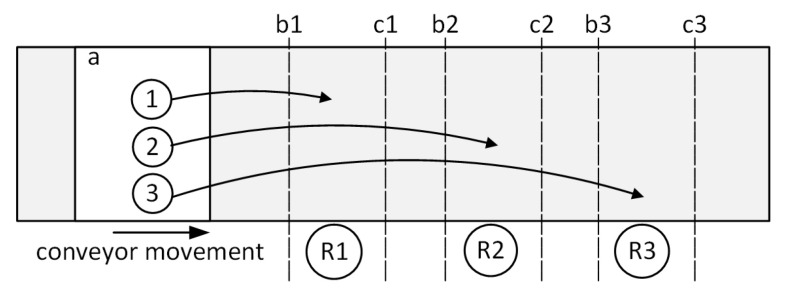
General view of distribution of items to selected Work Areas—LB algorithm: a—image frame; 1, 2, 3—items; R1, R2, R3—robots; b1, b2, b3—WA enter; c1, c2, c3—WA exit.

In accordance with the expectations of the client and the requirements of the production process, the production efficiency of the robotic packing station was proposed and analyzed. The fulfillment of the requirements was realized using two sorting algorithms. In the first algorithm—load balancing (LB)—workpieces are distributed to different work areas (specific items are allocated to different robots). Figure 2 shows an example of items distribution for three robots. After the object is detected by the vision system, it is assigned to the selected work area (WA) operated by specific robot. The working range is limited by two parameters: WA enter and WA exit. WA enter is the limit from where the robot starts to execute item targets on the work area. WA exit is the limit from where the robot considers an item target as lost on the work area (Figure 2). In this case, item positions will be distributed among the work areas in a way to avoid sending two adjacent positions to the same work area.

**Figure 3:**
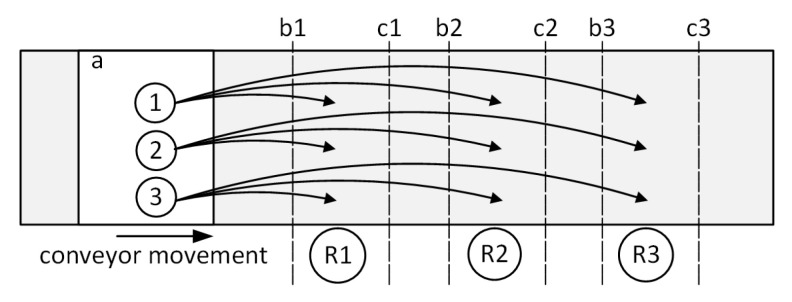
General view of distribution of items to selected Work Areas—ATC algorithm: a—image frame; 1, 2, 3—items; R1, R2, R3—robots; b1, b2, b3—WA enter; c1, c2, c3—WA exit.

The second algorithm—Adaptive Task Completion (ATC)—is based on the principle of distributing all objects detected by the vision system to all work areas (Figure 3). This means that the robots always have more objects available to handle compared to load balancing algorithm. In other words, it can be said that all items assigned to the ATC group are sent to every work area in the group, and the items that are not accessed by the first work area will be accessed by any of the other work areas. Figure 3 shows an example of items distribution for three. After the object is detected by the vision system, it is assigned to every work area of the group. Similarly, to the previous case, the work areas are limited by two parameters: WA enter and WA exit.

**Figure 4:**
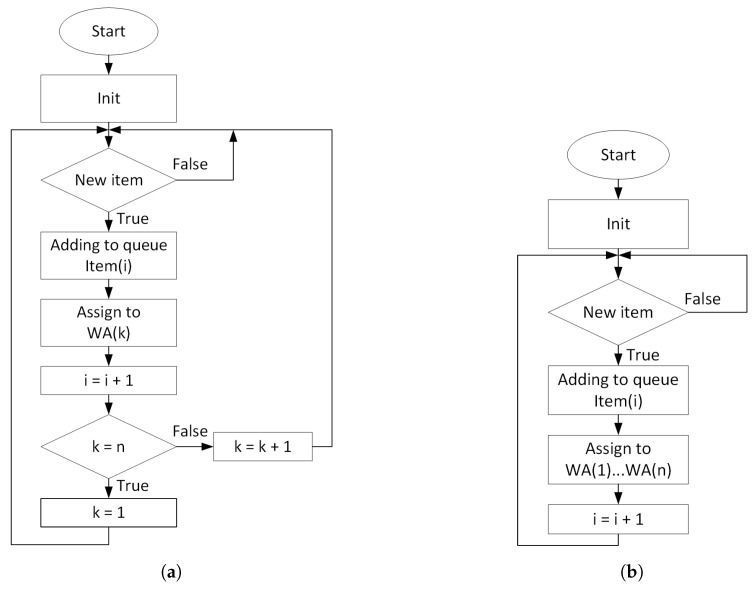
Distribution algorithms: (**a**) LB; (**b**) ATC.

Figure 4 shows the distribution algorithms of LB and ATC where:

- *i*—item index,
- *k*—work area index,
- *n*—number of work areas in the station.

Based on the presented algorithms, performance tests of the station were carried out, including the occurrence of possible emergency situations. Two possible types of emergencies have been chosen:

- the robot has halted its movement due to hardware or software error, without emergency stopping the robot and the station. If the position of the robot does not interfere with other working devices, it is possible to complete the production cycle without operator intervention,
- after the occurrence of a service or emergency stop, if the robot does not collide with other equipment, the operator can resume production, bypassing the defective machine, to complete the production cycle.

Maintaining production, in the case of such emergency states, may be of key importance, especially when restarting production after a longer downtime requires a lot of work, e.g., the need to carry out maintenance work, removing the remaining material, cleaning the machine, and performing complex start-up procedure (industrial furnaces and washers) and financial outlay. Such considerations are advisable especially in the case of the vision of production of Industry 4.0, where it is expected that production series will be short and products are highly customized, which may lead to a situation where the completion of production, even with limited efficiency, will be the most profitable solution.

Section 2 of the article presents a description of a robotic production station for packaging one type of product, developed in the RobotStudio environment. According to the process requirements, the station parameters were selected and implemented using Picking PowerPac and PickMaster 3. Section 3 contains the results of conducted simulation tests for two work algorithms—LB and ATC. On their basis, key station indicators were determined for defined scenarios. A novelty of the approach is the inclusion of emergency situations—in which selected robots were stopped. It can be used to evaluate algorithms and efficiency of the station. In Section 4, an analysis of the obtained results was carried out and conclusions were included.

## 2. Robotic Cell Description

**Figure 5:**
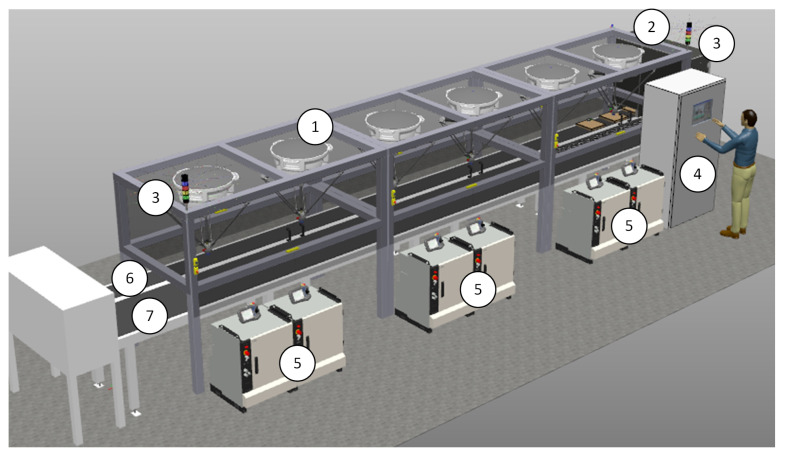
RobotStudio—general view: 1—IRB360 robots, 2—cameras, 3—light column, 4—control cabinet with HMI, 5—IRC5 controllers with FlexPendants, 6, 7—conveyors.

According to the client’s requirements, the digital twin station was built with the use of ABB IRB 360 robots. We used RobotStudio 2019 with the PickingPower Pac package to develop the virtual station (Figure 5). RobotStudio, which is built based on Virtual Controller (an exact copy of the IRC5 controller software), allows us to perform realistic simulations, in which we used configuration files from real robots. Control programs have been developed in RAPID programming language.

**Figure 6:**
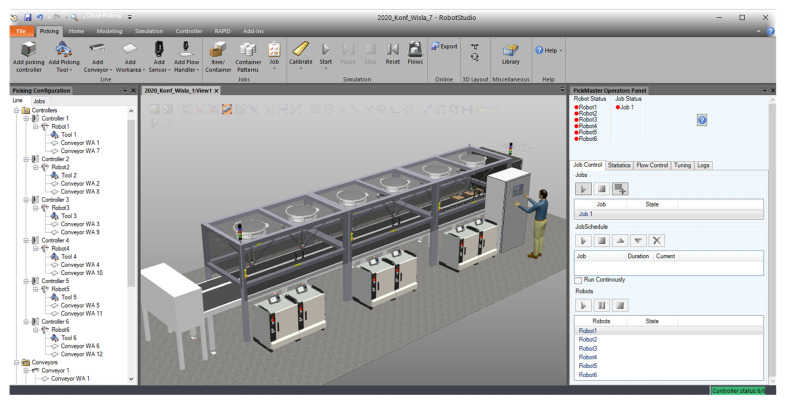
PickingPower Pac—general view.

**Figure 7:**
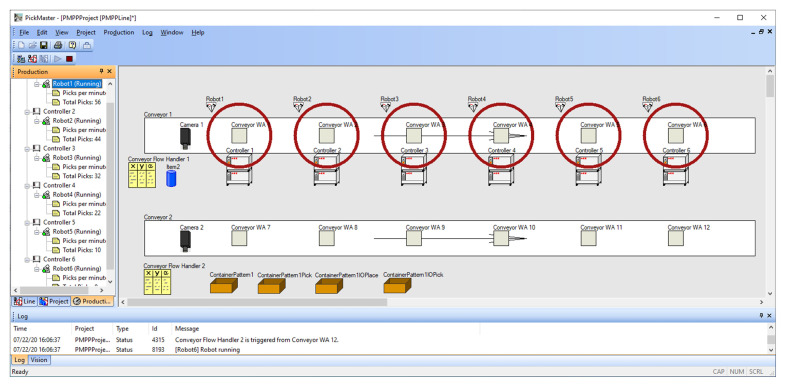
PickMaster3—Line and Project configuration.

PickingPower Pac (Figure 6) allowed us to create and test programs based on the PickMaster3 software (Figure 7). The use of such a software configuration made it possible to fully simulate the real conditions in which the PickMaster3 program is responsible for controlling the packaging application by providing information to robot controllers about the products and containers located on the conveyors.

### 2.1. Robotic Production Cell Components

The main components of the designed station are (Figure 5):

- six ABB IRB 360 robots with IRC5 controllers,
- three frames for robots,
- two cameras,
- two conveyors (Cnv1 and Cnv2),
- control cabinet.

#### 2.1.1. IRB360 Robot with an IRC5 Controller

**Figure 8:**
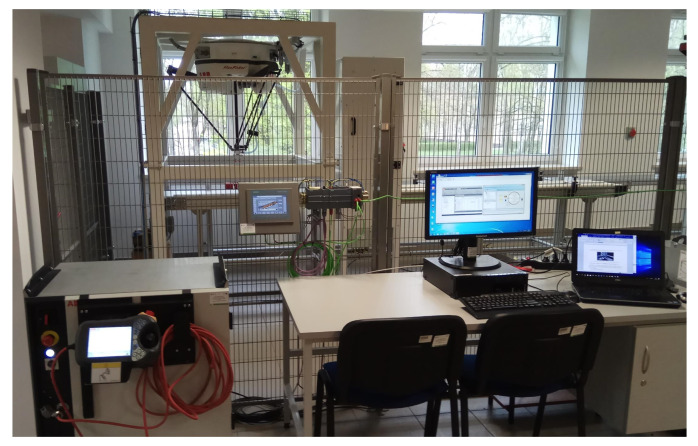
General view of IRB 360 FlexPicker with IRC 5 controller.

We used IRB 360 FlexPicker robots (Figure 8) with an acceleration 150 m/s${}^{2}$, max. speed of 10 m/s and a handling capacity of 1 kg. The robots are equipped with the IRC5 controller and robot control software—RobotWare. The IRC5 controller contains the electronics required to control the manipulator and peripheral equipment. In our station, we proposed six single cabinet controllers equipped with FlexPendants.

#### 2.1.2. Cameras

**Figure 9:**
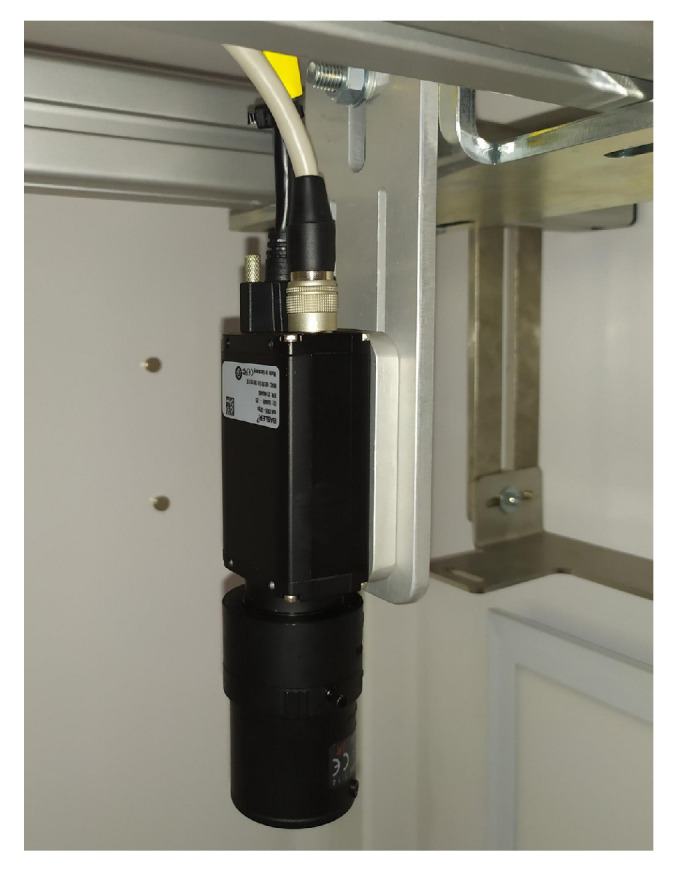
General view of Scout scA1300-32gc camera with Tamron lens.

Basler scA1300-32gc cameras with a resolution of 1294 × 964 pixels and an acquisition speed of 32 frames per second were proposed for the robotic cell. Tamron aspherical lenses with variable focal length (10–40 mm) were selected for the cameras (Figure 9).

#### 2.1.3. Conveyors (Cnv1 and Cnv2)

The station is equipped with two belt conveyors (Figure 5) with the following parameters:

- conveyor Cvn1—length—10 m, height—1 m, Width—0.8 m,
- conveyor Cvn2—length—10 m, height—1 m, Width—0.4 m.

#### 2.1.4. Control Cabinet

The control cabinet with the HMI is responsible for controlling the entire production process. It contains programmable logic controller and a safety controller.

### 2.2. Robotic Production Cell Parameters

To meet process requirements, we had to set:

- robot’s movement parameters,
- items and containers flow and patterns,
- conveyors properties,
- distribution settings,
- conveyors flow handlers’ properties,
- conveyor work area (WA) properties.

Industrial robot parameters and software options were taken from a backup of a real robot that is used in Robotics Laboratory at Military University of Technology in Warsaw (Figure 8). As a result, it can be said that the prepared station, equipped with virtual controllers is a digital twin of a real robotic cell. According to the producer, such technology offers 99% replication of the actual operations of the physical equipment. Because of that, we could test, analyze, and optimize the performance of the proposed station without using real equipment. Creating a virtual station can reduce the cost of product development and implementation time. It can also save costs before deciding on investments. Achieving the process performance required setting robot speed to 10 m/s and acceleration to 150 m/s${}^{2}$.

**Figure 10:**
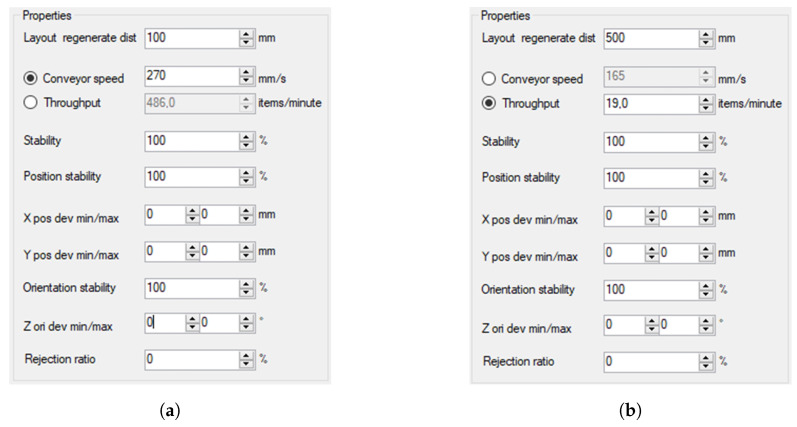
Flow properties window: (**a**) item; (**b**) container.

In the next step, we defined flow parameters of items and containers (Figure 10). Flow is used to define how they are generated in simulation.

Figure 10 shows flow parameters for generated items and containers. Parameters are as follows:

- Layout regeneration data—describes the distance between generated items,
- Conveyor speed—desired conveyor speed in mm/s,
- Throughput—desired item throughput when using the flow,
- Stability—probability of item generation,
- Position stability—probability of item generation on correct position,
- X/Y pos dev min/max—defines minimum and maximum deviation from correct position,
- Orientation stability—probability of item generation in correct orientation,
- Z on dev min/max—defines minimum and maximum deviation from correct orientation,
- Rejection ratio—defines the probability that a generated item becomes rejected by a camera.

We defined conveyors motion properties:

- acceleration—0.5 m/s${}^{2}$,
- deceleration—1 m/s${}^{2}$,
- speed—changed during the tests, as shown in the Results section.

In order to define to which work area the item shall be distributed for pick and place process, a distribution setting had to be set. There are four basic options: Work Area, Bypass, Load Balance (LB), and Adaptive Task Control (ATC). It is also possible to change rebalance strategy, from either never rebalancing to rebalancing among running and paused work areas.

Properly setting a flow handler requires determining a sensor trigger distance and sensor position filter. The first parameter is responsible for the distance between consecutive recording of the camera. In case it’s too long, some of the items may not be detected. A position filter describes a distance between two detected objects. If it’s too low, some of the objects may be counter as one. For our process, the sensor trigger distance was set to 150 mm and the sensor position filter to 0 mm. These values allowed us to achieve the required efficiency.

Conveyor work areas are divided into two groups: WA 1–6 is responsible for picking the items from conveyor Cnv1, while WA 7–12 is responsible for placing the items into containers on conveyor Cnv2.

For the WA 1–6 group, the parameters set is as follows:

- Pick elevation—vertical distance, from where robot approaches the target—30 mm,
- Pick time—time the robot tracks along the conveyor—0.035 s,
- Vacuum activation—time before or after reaching the target when vacuum is set— −0.025 s,
- Enter—limit from where the robot starts to execute item targets— −250 mm,
- Exit—limit from where robot considers item targets as lost—100 mm.

For the WA 7–12 group, the parameters set is as follows:

- Pick elevation—vertical distance, from where robot approaches the target—30 mm,
- Place time—time the robot tracks along the conveyor—0.035 s,
- Vacuum reversion—time before or after reaching half of the place time, blow is set— −0.025 s,
- Vacuum off—time before or after reaching half of the place time, vacuum is reset— −0.025 s,
- Enter—limit from where the robot starts to execute item targets— −300 mm,
- Exit—limit from where robot considers item targets as lost—100 mm.

### 2.3. Software Implementation of the Station

Control programs of the robots were prepared in RAPID programming language. They were divided into routines (for example pickplace sequence, Service, HomePos), so they are clear, flexible, and easily adjustable. The robot controller is receiving item coordinates from the PickMaster system. Indexed and queued items are picked and placed by each robot.

Pick and place application in our project consists of two parts (Figure 6):

- Line—contain all the physical objects—two cameras, two conveyors, and six IRB360 robots with IRC5 controllers,
- Project—contain items to be picked, connections between WA, items, and cameras—twelve work areas, item, two flow handlers, and one container pattern.

**Figure 11:**
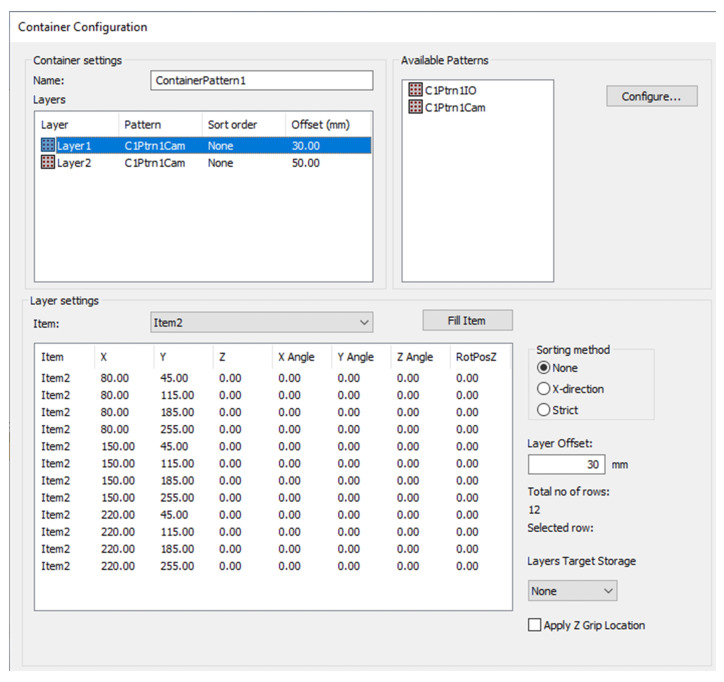
Container configuration view.

According to the client requirements, the container should contain two layers of items. There should be 12 items on each layer. In order to conduct the research, a container and a detail model were defined. Figure 11 shows a configuration of a container pattern.

## 3. Results and Discussion

A product packaging line equipped with six delta industrial robots and two conveyors (input—Cnv1, and output—Cvn2) was tested according to the previously presented assumptions.

### 3.1. Test 1

In the first test, the influence of the speed of Cnv1 and Cnv2 conveyors on the number of completed containers was investigated. We considered the minimization of the number of overflown items for LB and ATC algorithms, without the necessity to stop the conveyors. Moreover, the pick rate of individual robots for both packaging algorithms was verified by determining their work cycles in a unit of time. Each test lasted 400 s, with the first 100 s, taken as the time needed for filling the line. The test was conducted for the parameters shown in Figure 10 and Figure 11.

For the assumed station efficiency, conveyor speed Cnv2 = 130 mm/s (Figure 10b—Conveyor speed). By changing the Cnv1 speed (Figure 10a—Conveyor speed), the effect of changing the Cnv1 speed on the use of specific robots in the packaging process for LB and ATC algorithms was checked. Twelve tests were performed for each algorithm (LB and ATC); each test time was 400 s. The results from the period 0–100 s were rejected (station fill-up).

For the LB algorithm, the average pick rate for 12 attempts was 375.48 min${}^{-1}$. For individual robots, the average pick rate was as follows:

Robot 1 (R1) = 62.57 min${}^{-1}$, R2 = 62.57 min${}^{-1}$, R3 = 62.53 min${}^{-1}$, R4 = 62.61 min${}^{-1}$, R5 = 62.63 min${}^{-1}$, R6 = 62.57 min${}^{-1}$ (Table 1).

For the ATC algorithm, the average pick rate for 12 attempts was 376.12 min${}^{-1}$. For individual robots, it was as follows:

R1 = 78.37 min${}^{-1}$, R2 = 78.40 min${}^{-1}$, R3 = 107.47 min${}^{-1}$, R4 = 88.05 min${}^{-1}$, R5 = 23.82 min${}^{-1}$, R6 = 0.02 min${}^{-1}$ (Table 1).

The speed of all robots in the test has been set to 10,000 mm/s. The results presented above show that the robots are not used in 100%. This is especially visible for the ATC algorithm. The R5 and R6 robots performed fewer production cycles because the parts were distributed simultaneously to all work areas. Therefore, robots, R1, R2, R3, and R4 were able to complete containers by taking more items. The LB algorithm allocates items to specific work areas (evenly between all robots), so the pick rates on all robots are the same. For the presented station configuration, it would be advisable to select the optimum maximum speed of the robots in order to load them evenly.

For the LB (items distributed to specified work areas) and ATC (items distributed simultaneously to all work areas) algorithms, the maximum speed of all robots was limited to 3000 mm/s (according to the manufacturer’s data, this ensures longer trouble-free operation). The packaging process continued to be carried out correctly, i.e., all containers were completed. In addition, the ATC algorithm allowed for the introduction of different speeds of specific robots, i.e., less or more pick rate of specific machines. The LB algorithm ensures even wear of machines. The ATC algorithm, depending on preferences, gives the possibility of even load distribution (it is not as accurate as LB), or allows for reducing pick rate for selected robots while increasing it for others.

For the assumed Cnv1 speed (Figure 10b—Conveyor speed = 210 mm/s)—corresponding to the performance of the machine producing the items—by changing the Cnv2 speed (Figure 10a—Conveyor speed) the maximum line efficiency for the assumed configuration and the use of specific robots in the packaging process for LB and ATC algorithms was checked.

The results obtained indicate that, for a Cnv1speed of 210 mm/s, the maximum Cnv2 speed for which all containers have been completed is 131 mm/s. According to the table above (Table 2), even a small increase of Cnv2 speed, the number of uncompleted containers increased very quickly for both algorithms. A change of 1 mm/s caused a decrease in the number of completed containers by several percent.

Determining the maximum efficiency of the station for LB and ATC algorithms, assuming the maximum number of containers and minimizing the number of overflown items is possible considering the results obtained in points 1 and 2. In this test, the speeds of both conveyors were changed (Figure 10—Conveyor speed).

The selection of Cvn1 and Cvn2 speed was aimed at maximizing of station efficiency for LB and ATC algorithms, assuming the completion of the maximum number of containers and minimizing the number of overflown items during continuous operation. The tables (Table 3 and Table 4) present the statistics related to picking and placing items.

According to the results presented in the tables (Table 5 and Table 6), it can be stated that, for the tested station configuration:

- LB algorithm (Table 5)—the station achieved the maximum efficiency of 1188 completed containers per hour (no uncompleted containers). This value was obtained for conveyor speed: (Cnv1 = 270 mm/s, Cnv2 = 165 mm/s). Moreover, for these parameters, the requirement of maximum 5% of the overflown items was met (2.22%).
- ATC algorithm (Table 6)—the station obtained maximum efficiency of 1380 completed containers per hour (no uncompleted containers). This value was obtained for conveyor speed: (Cnv1 = 330 mm/s, Cnv2 = 195 mm/s). However, for these parameters, the requirement of maximum 5% of overflown items was not met (5.43%). Therefore, it was necessary to reduce the conveyor speed (Cnv1 = 310 mm/s, Cnv2 = 185 mm/s). In this case, the efficiency of 1332 completed containers per hour was obtained (no uncompleted containers), and the percentage of overflown items reached 4.55%.

**Figure 12:**
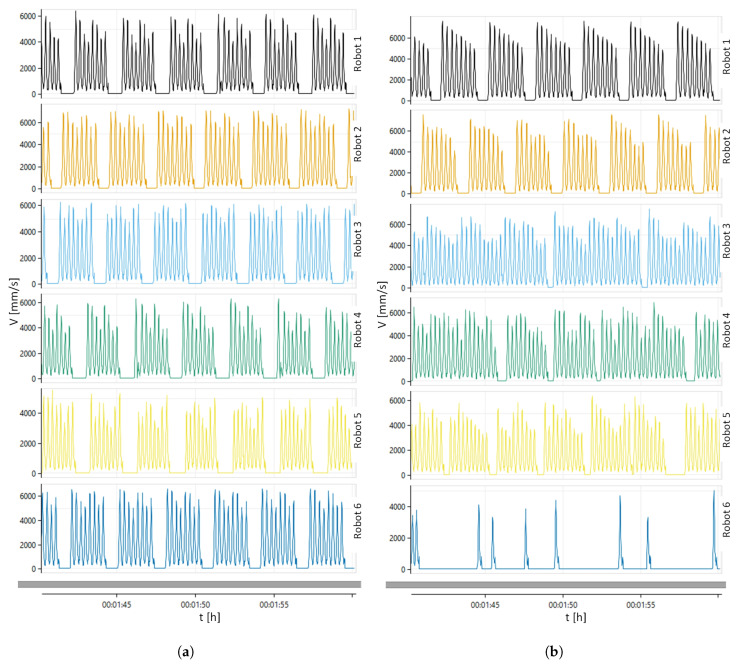
TCP (Tool Center Point) speed: (**a**) LB algorithm; (**b**) ATC algorithm.

Figure 12 shows the TCP speed graphs of packaging robots. For the LB algorithm (Figure 12a), the maximum speeds for specific robots are as follows: R1 = 6450 mm/s, R2 = 7500 mm/s, R3 = 6500 mm/s, R4 = 6375 mm/s, R5 = 5500 mm/s, R6 = 7200 mm/s. Due to the balanced distribution of products to specific work areas, each robot performs the same number of operations. For the ATC algorithm (Figure 12b), the maximum speeds for specific robots are as follows: R1 = 7500 mm/s, R2 = 7500 mm/s, R3 = 7200 mm/s, R4 = 7000 mm/s, R5 = 6450 mm/s, and R6 = 6300 mm/s. Due to the simultaneous distribution of products to all work areas, the robots perform a different number of operations. As can be seen (Figure 12a,b) on robots 3 and 4, which perform more operations for the ATC algorithm, and robot 6, which performs fewer operations for the ATC algorithm.

### 3.2. Test 2

**Figure 13:**
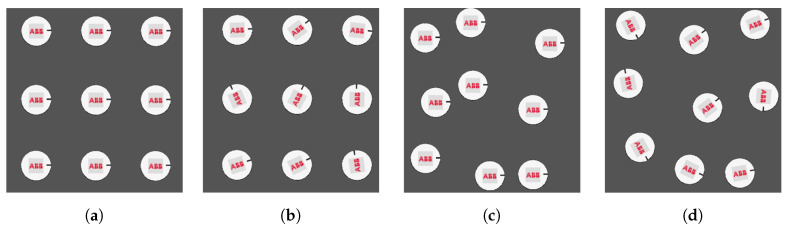
Patters of items: (**a**) stable position and orientation; (**b**) random orientation; (**c**) random position; (**d**) random orientation; and position.

In the second test, the influence of random position and orientation of items (Figure 13) on the efficiency of LB and ATC algorithms was tested. Conveyor speeds Cnv1 = 270 mm/s and Cnv2 = 165 mm/s were assumed. For these speeds, 100% of completed containers was obtained for both algorithms (LB and ATC).

**Figure 14:**
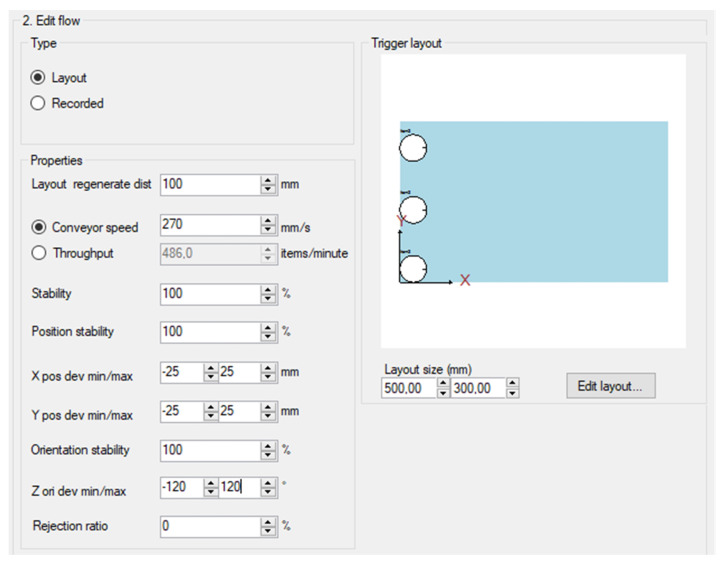
Item flow properties’ window.

Testing of the effect of random item position on the performance of LB and ATC algorithms. The measurements were made for different values of Position stability parameter (0%, 25%, 50%, 75%, 100%) (Figure 14).

The study showed that the ATC algorithm, for which a higher efficiency was obtained compared to the LB algorithm for an orderly distribution of details, is more sensitive to the position stability parameter (Table 7 and Table 8). The LB algorithm does not show such sensitivity.

Testing of the effect of random item orientation on the performance of LB and ATC algorithms. The measurements were made for different values of Orientation stability parameter (0%, 25%, 50%, 75%, 100%).

The study showed that a change in the orientation of the items does not significantly affect the performance of the station regardless of the algorithm used (Table 9 and Table 10).

Testing of the effect of random item orientation and position on the performance of LB and ATC algorithms. The measurements were made for different values of orientation stability parameter (0%, 25%, 50%, 75%, 100%) and Position stability parameter (0%, 25%, 50%, 75%, 100%).

The study showed (Table 11 and Table 12) that the combination of randomly generated position and orientation of the items gives a very similar effect to the first case (Table 7 and Table 8). This is mainly due to the distance the robot makes during the packaging process.

### 3.3. Test 3

Based on the results obtained, the optimum speed for the assumed process was determined—Cnv1—270 mm/s, Cnv2—165 mm/s. Emergency tests were carried out for the case where the items orientation and position were randomly changing, which is the most demanding case for a robotic packing station for one product.

Emergency situation, of at least one robot, for the LB algorithm has shown that none of the containers have been completed. The LB algorithm does not allow automatic continuation of production without the intervention of a robot programmer in the form of reconfiguration of lines (hardware) and project (software) of the robotic station. In this case, it is necessary to stop production until the failure is fixed. Therefore, the LB algorithm does not meet the requirements set for the process and cannot be used in case of a possible emergency.

Due to the characteristics of the ATC algorithm, in which all items are distributed simultaneously to multiple work areas, it is possible to adapt the station to changing production conditions and ensure efficiency in case of an emergency.

Table 13 presents the results of computer simulations carried out for assumptions:

- position stability 0% and Orientation stability 0%,
- number of working industrial robots from 4 to 6,
- change of transporter speed in the range Cnv1—265–260 mm/s and Cnv2—160–155 mm/s.

As shown in Table 12, reaching the assumed efficiency was realized for the ATC algorithm for Cnv1—270 mm/s, Cnv2—165 mm/s. However, in the case of emergency, the percentage of container completion (Position stability 0% and Orientation stability 0%) was 89.90%, which does not meet the requirements (max 5%). Therefore, it was necessary to lower the speed of the conveyors to perform emergency tests (Table 13).

At the assumed speeds, the efficiency was obtained at the level of 1140 completed containers per hour, reaching 99% of completion. In the event of an emergency—stopping of one of the industrial robots, the efficiency dropped to 1068 completed containers per hour, reaching 94% of completion. The obtained efficiency in an emergency is 93% of the obtained performance for six robots. In case of the stopping of another robot—the work of four robots, the efficiency decreased to 0% (each container was missing 1 to 6 pieces).

For the presented situation, it is possible to maintain production at the level of 93% of the assumed efficiency in case of failure of one robot. Each subsequent failure makes it impossible to continue with production that meets the requirements.

Because 94% of the completion was achieved in an emergency, additional tests were carried out to determine the optimal speed of transporters, with requirements to fill all containers. Full completion was achieved for conveyor speeds—Cnv1—260 mm/s and Cnv2—155 mm/s (Table 13). For these parameters, efficiency of 1116 containers per hour was obtained for both six and five robot configurations. Despite the decrease of conveyor speed, the achieved efficiency for an emergency (one industrial robot stopped) increased from the initial efficiency (1068 containers per hour) to 1116 containers per hour. This is due to a higher percentage of completed containers (100 to 94%). An attempt to stop two robots (4 running) resulted in 0 completed containers.

## 4. Conclusions

Thanks to digital twin technology, it is possible to prepare virtual robotic cells that replicate psychical devices and controllers with all available options and functions. It allows users to conduct comprehensive tests, including analyzing robot speed, acceleration and pick rates, conveyor speed, and overall station efficiency.

Results of extensive testing using the virtual station were shown in Section 3. First, a test was conducted to check the differences between pick rates of each individual robot, while using the LB and ATC sorting algorithm. Data from Table 1 show that industrial robots are not fully used; it is especially visible with ATC algorithm, where robots 5 and 6 have done less cycles than the rest. Pick rates obtained for these robots were 23.82 min${}^{-1}$ and 0.02 min${}^{-1}$ as opposed to pick rates of robots 1–4 (R1 = 78.37 min${}^{-1}$, R2 = 78.40 min${}^{-1}$, R3 = 107.47 min${}^{-1}$, R4 = 88.05 min${}^{-1}$). These results are similar to those of the noncooperative dynamic game method, where, if an item is not picked by a robot, it is transferred to a second one. The station is able to achieve almost 100% of the picked items, with an increase in the number of robots not leading to an increase in efficiency, but some of the machines spend more idle time [35]. These differences can lead to uneven mechanical wear of machines; since they work as a group, this phenomenon is undesirable because it can lead to irregular service cycles. More frequent services generate additional costs for the user.

The prepared station allows for determining maximum efficiency for assumed input conditions. In our case, the speed of Cnv1 (corresponding to the number of distributed items) was set to 210 mm/s. Table 2 show results of tests for increasing Cnv2 speed (131–140 mm/s). Both algorithms netted 100% container completion for Cnv1 = 210 mm/s and Cnv2 = 131 mm/s. With an increase in Cnv2 speed, the percentage of completed containers dropped significantly.

In Table 3, Table 4, Table 5 and Table 6, we showed results of the experiment in which we obtained the value of maximum efficiency, by changing the speeds of both conveyors. Results for LB were 1188 completed containers per hour; it was achieved for Cnv1 = 270 mm/s and Cnv2 = 165 mm/s. For these speeds, a percentage of overflown items was 2.22%, which is lower than maximum of 5% as in project requirements. Results for ATC were 1332 completed containers per hour; it was achieved for Cnv1 = 310 mm/s and Cnv2 = 185 mm/s. For these speeds, the percentage of overflown items was 4.55%, which is lower than the maximum of 5% as in project requirements—meaning that the ATC algorithm can handle more containers (at higher speed of the conveyors), but, at the same time, the number of overflown items increased.

The article also presents the results of tests carried out for emergency situations described in Section 1. For the LB algorithm, all tests carried out, regardless of the process input parameters, were negative. None of the produced containers were fully filled (each container was missing 1 to 6 pieces), so they should be treated as scraps. For the ATC algorithm, it was possible to continue production after an emergency situation. Depending on the feeder speed (Table 13), a production efficiency of 94% to 98% of the maximum station efficiency was achieved. The appropriate flow of products in multi-robot systems depends on the configuration of the station; each hardware change (e.g., halting the robot) leads to the need to change the part-dispatching rules [54]. The applied ATC algorithm enables automatic adjustment of control strategy—by changing the part-dispatching rules, so that the item flow of the station remains at the same level even during emergency situation.

The developed station allows for conducting repeatable simulations and gives the possibility of introducing changes in the input parameters of the production process. Prepared robot programs are easily modifiable and can be adjusted to the user’s needs. This makes it possible to study the impact of individual process parameters on the functioning of the station and on its efficiency, robot cycle times, and the number of handled and rejected items.

## Figures and Tables

**Table 1:** Pick Rate for LB i ATC algorithms—Cnv2 = 130 mm/s.

Nb	Cnv1 [mm/s]	Cnv2 [mm/s]	Conveyor WA 1		Conveyor WA 2		Conveyor WA 3		Conveyor WA 4		Conveyor WA 5		Conveyor WA 6	
			LB	ATC	LB	ATC	LB	ATC	LB	ATC	LB	ATC	LB	ATC
1	210	130	62.4	78	62.4	78	62.4	108.8	62.4	92.2	62.4	16.8	62.4	0.2
2	215	130	62.4	78	62.4	78	61.6	108.4	62.4	93.2	62.4	16.4	62.4	0
3	220	130	62.4	78.6	62.4	78.8	62.4	109.2	62.4	94.2	62.4	17.2	62.4	0
4	225	130	62.4	78	62.4	78	62.4	106.6	62.4	89.4	62.4	22.4	62.4	0
5	230	130	61.8	78.8	61.8	78.8	61.8	107.8	61.8	86.6	61.6	26	62	0
6	240	130	62.8	78	62.8	78	63	106.8	63	89.6	63.2	22.2	63	0
7	250	130	63	78	63	78	63	106.6	63.2	86	63.2	26	62.8	0
8	260	130	63	78	63	78	63	107.2	63	88.2	63.2	22.6	62.8	0
9	270	130	62.8	78.8	63	78.8	63	107.6	62.4	88.6	63.2	24	62.8	0
10	280	130	62.4	78.6	62.4	78.6	62.4	107.6	62.4	83.4	62.4	29.4	62.4	0
11	290	130	63	78.8	62.8	78.8	63	106.4	63.6	83.4	62.8	30.8	63	0
12	300	130	62.4	78.8	62.4	79	62.4	106.6	62.4	81.8	62.4	32	62.4	0

**Table 2:** Container completion percentage.

Nb	Cnv1 [mm/s]	Cnv2 [mm/s]	LB			ATC		
			Completed	Uncompleted	% Completed	Completed	Uncompleted	% Completed
1	210	131	79	0	100.00	78	0	100.00
2	210	132	75	4	94.94	69	9	88.46
3	210	133	57	23	71.25	56	23	70.89
4	210	134	53	27	66.25	44	36	55.00
5	210	135	34	48	41.46	33	49	40.24
6	210	140	22	62	26.19	7	77	8.33

**Table 3:** Items handling statistics—LB algorithm.

Nb	Cnv1 [mm/s]	Cnv2 [mm/s]	Pick Rate						Completed	Uncompleted	% Completed
			Conveyor WA 1	Conveyor WA 2	Conveyor WA 3	Conveyor WA 4	Conveyor WA 5	Conveyor WA 6			
1	220	135	64	64.8	64.6	65.4	64.8	64.8	1946	37	98.13
2	230	140	67.2	67.2	67.2	67.2	67.2	67.2	2016	144	93.33
3	240	145	69.6	69.6	69.6	69.6	69.6	69.6	2088	72	96.67
4	250	150	72	71.8	72	72	72	72	2159	90	96.00
5	260	155	74.4	74.4	74.6	74.4	74.4	74.4	2233	108	95.39
6	270	160	79.2	79.2	79.2	79.2	79.2	79.2	2376	54	97.78
7	280	165	80.8	80.8	79.2	80.6	80.8	80.8	2415	76	96.95
8	290	170	84.8	84.8	82.6	85	84.8	84.8	2534	103	96.09
9	300	175	86.4	86.4	70.2	86.4	86.4	86.4	2511	189	93.00

**Table 4:** Items handling statistics—ATC algorithm.

Nb	Cnv1 [mm/s]	Cnv2 [mm/s]	Pick Rate						Completed	Uncompleted	% Completed
			Conveyor WA 1	Conveyor WA 2	Conveyor WA 3	Conveyor WA 4	Conveyor WA 5	Conveyor WA 6			
1	220	135	80.2	80.2	107	87.2	30	0.2	1924	36	98.16
2	230	140	84.8	85	111.2	85.8	42.6	0	2047	54	97.43
3	240	145	87.8	87.8	109	88	48	0	2103	72	96.69
4	270	165	79.2	79.2	105	96.8	95.6	19.2	2375	55	97.74
5	280	170	81.6	81.6	102.2	93.4	89.4	41	2446	73	97.10
6	290	175	84.8	84.8	106.2	91.2	87.6	54.8	2547	91	96.55
7	300	180	86.4	86.4	108	87.2	86.4	64	2592	108	96.00
8	310	185	88.8	88.8	102.8	97	88.8	66.6	2664	127	95.45
9	320	190	91.2	91.2	102.2	102	91.2	68.8	2733	146	94.93
10	330	195	92.8	92.6	103.4	104.6	92.6	71.4	2787	160	94.57
11	340	200	96	96	102.2	105.2	96	79.8	2876	185	93.96
12	350	205	97.4	97.4	102.8	104	97.4	83.2	2911	209	93.30
13	360	210	100.8	100.8	102.6	103.4	96.4	92.6	2983	256	92.10
14	370	215	97.8	97.8	101	103.8	94	102.2	2983	353	89.42

**Table 5:** Containers handling statistics—LB algorithm.

Nb	Cnv1 [mm/s]	Cnv2 [mm/s]	Completed	Uncompleted	% Completed	Efficiency [h${}^{-1}$]
1	220	135	81	0	100	972
2	230	140	84	0	100	1008
3	240	145	87	0	100	1044
4	250	150	90	0	100	1080
5	260	155	93	0	100	1116
6	270	160	99	0	100	1188
7	280	165	96	4	96	1152
8	290	170	94	12	89	1128
9	300	175	25	83	23	300

**Table 6:** Containers handling statistics—ATC algorithm.

Nb	Cnv1 [mm/s]	Cnv2 [mm/s]	Completed	Uncompleted	% Completed	Efficiency [h${}^{-1}$]
1	220	135	80	0	100	960
2	230	140	85	0	100	1020
3	240	145	88	0	100	1056
4	270	165	98	1	99	1176
5	280	170	102	0	100	1224
6	290	175	105	0	100	1260
7	300	180	108	0	100	1296
8	310	185	111	0	100	1332
9	320	190	112	2	98	1344
10	330	195	115	0	100	1380
11	340	200	115	5	96	1380
12	350	205	109	13	89	1308
13	360	210	89	37	71	1068
14	370	215	63	66	49	756

**Table 7:** LB algorithm efficiency for random item positions.

Cnv1	Cnv2	Pos	Orient	LB			
				Completed	Uncompleted	% Completed	Efficiency [$h^{-1}$]
270	165	0	100	99	0	100	1188
270	165	25	100	100	0	100	1200
270	165	50	100	99	0	100	1188
270	165	75	100	97	2	97.98	1164
270	165	100	100	99	0	100	1188

**Table 8:** ATC algorithm efficiency for random item positions.

Cnv1	Cnv2	Pos	Orient	ATC			
				Completed	Uncompleted	% Completed	Efficiency [$h^{-1}$]
270	165	0	100	92	7	92.93	1104
270	165	25	100	95	4	95.96	1140
270	165	50	100	97	2	97.98	1164
270	165	75	100	92	7	92.93	1104
270	165	100	100	99	0	100	1188

**Table 9:** LB algorithm efficiency for random item orientations.

Cnv1	Cnv2	Pos	Orient	LB			
				Completed	Uncompleted	% Completed	Efficiency [h${}^{-1}$]
270	165	100	0	99	0	100	1188
270	165	100	25	100	0	100	1200
270	165	100	50	99	0	100	1188
270	165	100	75	99	0	100	1188
270	165	100	100	99	0	100	1188

**Table 10:** ATC algorithm efficiency for random item orientations.

Cnv1	Cnv2	Pos	Orient	ATC			
				Completed	Uncompleted	% Completed	Efficiency [h${}^{-1}$]
270	165	100	0	99	0	100	1188
270	165	100	25	99	0	100	1188
270	165	100	50	98	1	98.99	1176
270	165	100	75	96	3	96.97	1152
270	165	100	100	99	0	100	1188

**Table 11:** LB algorithm efficiency for random items positions and orientations.

Cnv1	Cnv2	Pos	Orient	LB			
				Completed	Uncompleted	% Completed	Efficiency [h${}^{-1}$]
270	165	0	0	99	0	100	1188
270	165	25	25	100	0	100	1200
270	165	50	50	99	0	100	1188
270	165	75	75	98	1	98.99	1176
270	165	100	100	99	0	100	1188

**Table 12:** ATC algorithm efficiency for random items’ positions and orientations.

Cnv1	Cnv2	Pos	Orient	ATC			
				Completed	Uncompleted	% Completed	Efficiency [h${}^{-1}$]
270	165	0	0	89	10	89.90	1068
270	165	25	25	95	4	95.96	1140
270	165	50	50	95	4	95.96	1140
270	165	75	75	96	3	96.97	1152
270	165	100	100	99	0	100	1188

**Table 13:** ATC algorithm efficiency in emergency situations.

Cnv1	Cnv2	Nb of Robots	Items Used	Items Overflow	Completed	Uncompleted	% Completed	Efficiency [h${}^{-1}$]
265	160	6	2303	83	95	1	98.96	1140
265	160	5	2304	85	89	6	93.68	1068
265	160	4	1674	737	0	95	0	0
260	155	6	2232	107	93	0	100	1116
260	155	5	2230	109	93	0	100	1116
260	155	4	1625	715	0	93	0	0
